# Primary Screening for Cervical Cancer Based on High-Risk Human Papillomavirus (HPV) Detection and HPV 16 and HPV 18 Genotyping, in Comparison to Cytology

**DOI:** 10.1371/journal.pone.0119755

**Published:** 2015-03-20

**Authors:** Theodoros Agorastos, Kimon Chatzistamatiou, Taxiarchis Katsamagkas, George Koliopoulos, Alexandros Daponte, Theocharis Constantinidis, Theodoros C. Constantinidis, and the HERMES study group

**Affiliations:** 1 4th Department of Obstetrics and Gynecology, Aristotle University of Thessaloniki, Hippokratio General Hospital, Thessaloniki, Greece; 2 3rd Department of Obstetrics and Gynecology, University of Athens, Attikon Hospital, Athens, Greece; 3 Department of Obstetrics and Gynecology, University of Thessaly, General University Hospital, Larissa, Greece; 4 Peripheral Laboratory of Public Health, Hellenic Center for Disease Control and Prevention, Ministry of Health, Alexandroupoli, Greece; 5 Laboratory of Hygiene and Environmental Protection / Laboratory of Microbiology, Democritus University of Thrace, Alexandroupoli, Greece; Georgetown University, UNITED STATES

## Abstract

**Objectives:**

The objective of the present study is to assess the performance of a high-risk human papillomavirus (HR-HPV) DNA test with individual HPV-16/HPV-18 genotyping as a method for primary cervical cancer screening compared with liquid-based cytology (LBC) in a population of Greek women taking part in routine cervical cancer screening.

**Methods:**

The study, conducted by the “HEllenic Real life Multicentric cErvical Screening” (HERMES) study group, involved the recruitment of 4,009 women, aged 25–55, who took part in routine cervical screening at nine Gynecology Departments in Greece. At first visit cervical specimens were collected for LBC and HPV testing using the Roche Cobas 4800 system. Women found positive for either cytology or HPV were referred for colposcopy, whereas women negative for both tests will be retested after three years. The study is ongoing and the results of the first screening round are reported herein.

**Results:**

Valid results for cytology and HPV testing were obtained for 3,993 women. The overall prevalence of HR-HPV was 12.7%, of HPV-16 2.7% and of HPV-18 1.4%. Of those referred for colposcopy, cervical intraepithelial neoplasia grade 2 or worse (CIN2+) was detected in 41 women (1.07%). At the threshold of CIN2+, cytology [atypical squamous cells of undetermined significance (ASC-US) or worse] and HPV testing showed a sensitivity of 53.7% and 100% respectively, without change between age groups. Cytology and HPV testing showed specificity of 96.8% and 90.3% respectively, which was increased in older women (≥30) in comparison to younger ones (25–29). Genotyping for HPV16/18 had similar accuracy to cytology for the detection of CIN2+ (sensitivity: 58.5%; specificity 97.5%) as well as for triage to colposcopy (sensitivity: 58.5% vs 53.7% for cytology).

**Conclusion:**

HPV testing has much better sensitivity than cytology to identify high-grade cervical lesions with slightly lower specificity. HPV testing with individual HPV-16/HPV-18 genotyping could represent a more accurate methodology for primary cervical cancer screening in comparison to liquid-based cytology, especially in older women.

## Introduction

Widespread screening of women with the Papanicolaou test has led to a substantial decrease in incidence and mortality due to cervical cancer in countries where it has been systematically implemented [1,2]. However, cervical cancer screening programs based on cytology have yielded significantly poorer results in countries where screening was not implemented systematically, especially in developing countries (http://globocan.iarc.fr/Pages/fact_sheets_cancer.aspx). Low coverage and poor patient compliance, subjective interpretation of results, in conjunction with factors concerning public health and legal issues, are some of the possible reasons for the substandard results that have been noted in cytology-based screening [3].

During the recent decades consensus has been reached on the direct relationship between HPV infection and cervical carcinogenesis [4,5], and it has been demonstrated that HPV infection is a necessary condition for the development of pre-invasive and invasive cervical cancer [6,7]. Currently more than 200 HPV types have been identified and those infecting the cervix have been categorized according to their oncogenic potential as high-risk (HR), (i.e.) with sufficient evidence for the causation of cervical cancer (16 – the most potent type -, 18, 31, 33, 35, 39, 45, 51, 52, 56, 58, and 59), possible high-risk, (i.e.) with limited evidence for the causation of cervical cancer (26, 53, 66, 67, 68, 70, 73, 82), and not classifiable as to their carcinogenicity to humans (6, 11) [8].

As HR-HPV types are detected in more than 99% of invasive cancer cases and in the vast majority of high-grade pre-invasive cases, HPV detection may be a reasonable alternative as a screening test for the detection of precursor lesions that would progress to cancer if not treated. In fact, the detection of HR-HPV types is considered today, by many, as a better method of screening than cytology [9–15].

Several studies have demonstrated that HPV screening is more sensitive in the detection of high-grade transformative lesions and cancer than the Papanicolaou test [16–20], as well as in preventing mortality from this disease [15,21]. In addition, a negative result in HPV testing allows for a greater time interval before repeat screening becomes necessary as compared to a normal Papanicolaou test result, allowing for less frequent testing and increased patient compliance with screening programs [14,22,23].

The main disadvantage of HPV testing as a primary screening technique is the relatively high incidence of false positive cases (low specificity). This is because the majority of HR-HPV infections are transient and will not result in diseases, particularly among younger women. The risk of this occurring depends partly on the specific HPV type [24–26], but also on genetic [27] and/or environmental factors, such as tobacco smoking [28].

Therefore, the improvement of the performance of systematic cervical cancer screening that is initially based on HPV testing depends on the implementation of secondary triage techniques which, within the group of HPV-positive women, will identify that sub-group of women with an especially high risk of developing pre-invasive or invasive lesions. One of the approaches that has been proposed is the identification of the HPV type. Women infected with HPV16 and/or HPV18 are more likely to develop intermediate grade CIN (CIN2) or worse lesions than women infected by other high-risk genotypes [29]. According to the large Portland Kaiser Cohort study, the 10-year cumulative probability of lesions CIN3 or worse was 17.2% for HPV16+ women and 13.6% for HPV18+ women, but only 3.0% for women positive for high-risk HPV but negative for HPV16 or HPV18 [24]. Similar results were reported for the 16-year cumulative probability of the same cohort [30].

Finally, before the implementation of HPV testing as a primary tool in a country’s screening program, the particular method used for the detection of HPV DNA should be tested in the country’s setting, as performance might not be similar between tests and countries. Indeed a meta-analysis has shown that there is considerable heterogeneity between studies depending both on the geographic location of the study and the type of HPV test used [31]. Based on this, the “HERMES” (HEllenic Real life Multicentric cErvical Screening) study group has performed the present multicentric study in the framework of general-population preventive cervical cancer screening in Greece, having as primary objective to examine the effectiveness and the accuracy of the cobas HPV test (Roche Molecular Systems, Pleasanton, CA, USA) as a primary technique in comparison to the established cytological method. The secondary objective was the formulation and evaluation of various triage algorithms after two rounds of screening. Here the results from the first round of screening are presented.

This is the only multicentric study that assesses the performance of a U.S. Food and Drug Administration (FDA) approved HPV test in various University Hospitals, Public Hospitals and Family Centers in Greece.

## Materials and Methods

### Study Population

Women aged 25–55 attending routine cervical screening at the outpatient clinics of nine Gynecology Departments (2 in Athens, 4 in Thessaloniki, 1 in Larissa, 1 in Patras and 1 in Alexandroupolis) were asked to be enrolled in the study. Exclusion criteria were current pregnancy, current or previous history of CIN in the past five years, follow up for cytological abnormalities and hysterectomy.

### Study design

From April 2011 until September 2013 women attending regular preventive cervical cancer screening procedures were informed of the study rationale and purpose and asked to provide written informed consent. At each visit (screening round) samples were collected for initial cytological evaluation. After that an aliquot of the remaining sample was used for HPV DNA testing.

**Fig 1:**
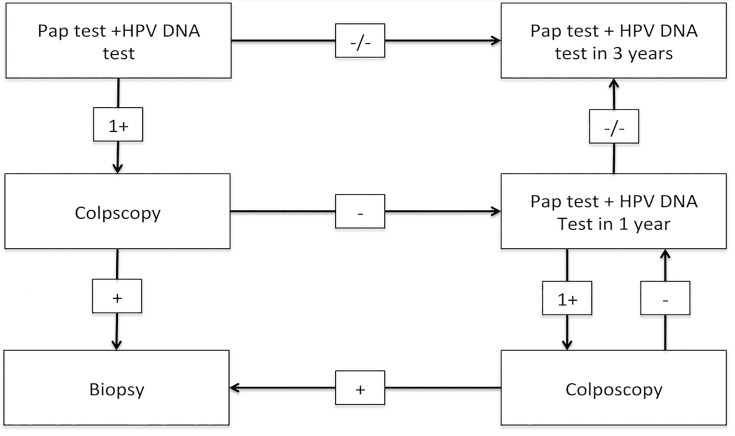
Flowchart of the study design. This article presents the results of the first screening round (cross-sectional phase) of a longitudinal screening test accuracy study.

The flowchart of the study design is presented in Fig. 1. The study design specifies that women with negative HPV DNA test and cytology results will be retested 3 years after the first visit. Women found positive either for cytology (ASCUS or worse) or HPV DNA testing (14HR HPV positive) undergo colposcopy examination in each OB/GYN Department involved. Upon the presence of abnormal colposcopic findings, multiple focal biopsies and/or endocervical curettage (ECC) should be taken. If there were no abnormal colposcopic findings the women will be recalled for HPV DNA testing and cytology examination one year after the initial testing. At the time of the second visit if HPV DNA testing and cytology are negative, women will be retested 3 years after the second visit, whereas if any of these tests is positive, colposcopy/biopsy will be performed.

### Cytology Testing

Samples were collected using the specially designed trident-shaped Cervex Brush (Rovers Medical Devices, B.V. Oss, The Netherlands) according to the manufacturer’s instructions. The brush was rotated five times in a clockwise direction taking a sample from the outer and inner cervix and cervical domes. After sample collection, the broom-type sampling device was directly immersed in the collection fluid (ThinPrep, Hologic, Bedford, MA, USA). The vial was hermetically sealed, and a pre-printed barcode tag was affixed for specimen identification. A fraction was used for cytology examination, while the remainder was transported within two weeks to the laboratory where HPV DNA testing was conducted.

Cytology smear examination was conducted at the corresponding cytology laboratories of the hospitals cooperating in the study as per usual practice. The cytologists were not aware of the HPV DNA test results, nor were the molecular biologists of the cytology results. Classification of the cytology results was conducted according to the Bethesda 2001 directive [32]. Positive findings, therefore fulfilling criteria for colposcopy referral, were considered as those classified as ASCUS or worse (ASCUS+).

### HPV DNA testing

Following cytology, an aliquot of the collected sample was transported within two weeks under a controlled temperature environment of 2–30°C to the Laboratory of Hygiene and Environmental Protection / Laboratory of Microbiology of the Democritus University of Thrace in Alexandroupolis, where HPV DNA testing was conducted. The collected samples were analysed for the detection of the genome of 14 high-risk Human Papilloma Viruses (HPV DNA testing) and for individual identification of HPV16/18, using the cobas HPV test by Roche.

The cobas 4800 HPV PCR master mix includes primers and fluorescently labeled probes to detect the DNA of 14 high-risk (HR) HPV types (16, 18, 31, 33, 35, 39, 45, 51, 52, 56, 58, 59, 66 and 68) and human beta-globin, which acts as a measure of human cellularity in the specimen. Specifically, the cobas HPV test employs primers that amplify a region of approximately 200 base pairs within the L1 polymorphic region of the HPV genome. The fluorescent signal from twelve HR types of HPV (12HR HPV) (31, 33, 35, 39, 45, 51, 52, 56, 58, 59, 66 and 68) is detected using the same fluorescent label, while the HPV 16, HPV 18 and beta-globin signals are detected with three separate spectrally unique fluorescent labels, respectively. The distinct individual wavelengths characterizing each label allow for simultaneous genotyping of HPV 16 and HPV 18 amplicon separately from the twelve other HR types.

### Colposcopy and histological examination

According to the study design, women found positive either for cytology or HPV DNA testing underwent colposcopy in each OB/GYN Department involved, by an experienced specialist. In addition, 106 randomly selected women with normal cytology findings and a negative HPV DNA test were referred for colposcopy (2.6% of the total of patients taking part in the first screening round). Upon the presence of abnormal or suspicious for invasion colposcopic findings -ASSIST colposcopy Code 1–3 [33] corresponding to the 2011 colposcopic terminology of the International Federation for Cervical Pathology and Colposcopy [34]-, multiple focal biopsies and/or ECC were taken from the abnormal area of the cervix. ECC was performed in case of invisible transformation zone. If the biopsy/ECC results were normal, the women were recalled for HPV DNA testing and cytology examination after one year. If the biopsy revealed CIN1 lesions, the women were re-examined after 6 months. In case the biopsy revealed CIN2 or worse (CIN2+) lesions, the patients were immediately referred for treatment. The primary endpoint of the study was the histological diagnosis of CIN2 or worse, and, as mentioned, such women were offered treatment and exited the study. After the treatment, when two different histopathology results had been obtained [i.e. from the biopsies/ECC and from the large loop excision of the transformation zone (LLETZ) or conisation], the more severe of the two results was considered as final diagnosis. Histology examination was conducted in each of the cooperating hospitals, and the pathologists were aware of the cytology and colposcopy result, but not of the HPV DNA test result. Pathological findings (CIN of any degree) were independently re-assessed by an independent specialized pathologist. If there was consensus in the diagnosis, the specimens were not re-examined. If consensus was not reached after the second examination, a third independent specialized pathologist examined the specimen in question so that at the end a two out of three decision was made.

### Statistical Analysis

The objective of screening is the detection of lesions CIN2 or worse. For 80% statistical validity and a statistical significance level p = 0.05, 44 cases of CIN2+ are required in order to detect reliably an increase in sensitivity from 75% to 96%. Using a calculated prevalence of 1.1% for CIN2+ [19,35], the required number of women would be approximately 4,000 per screening round.

The following diagnostic accuracy indices with 95% confidence intervals (CI) were calculated for LBC and HPV testing: Sensitivity, specificity, positive predictive value (PPV), negative predictive value (NPV), positive likelihood ratio (PLR) and negative likelihood ratio (NLR). For LBC two positivity thresholds were examined (ASCUS+ and LSIL+). From the HPV test results the accuracy indices of positivity for 14 types, of positivity for types 16 &18 and of type 16 only were calculated. The accuracy indices of the combination of positive LBC (ASCUS+, LSIL+ or HSIL+) and HPV 16 only and HPV16 or HPV 18 positivity were also calculated. Apart from the relative values of these variables, verification of the absolute specificity and sensitivity values was corroborated by random colposcopy of 2.6% of the women involved in the study, irrespective of their screening results.

All analyses were performed for the whole study population and separately for women 25–29 years and for women 30 years or over, as well as for women of different age groups. This was done in order to assess differences in diagnostic accuracy mainly for HPV testing in older versus younger women. The diagnostic accuracy indices between tests were compared on the basis of the overlapping of their 95% confidence intervals and with the McNemar’s test. Relative risks for harbouring CIN2+ were calculated for HPV test and cytology status using fourfold tables [36,37]. All analyses were performed with SPSS 22.0 statistical software (IBM Co., New York, United States).

### Ethics statement

The study protocol was approved by the Ethical Committee of the Aristotle University of Thessaloniki. (Protocol number A13719 / 31.08.2010). Our method involves the collection of data from anonymized consenting women. A patient data sheet is filled in by the physician/midwife for each woman enrolled. This sheet is accompanied by a patient information sheet, which every woman reads and signs giving her written informed consent. A unique barcode is then assigned to each participant, which accompanies her sample. If the woman chooses not to participate their encounter details are not recorded. The investigators are asked not to provide written consent to the research body, as this prevents recruited women from remaining anonymous.

## Results

The collaborating centres recruited 4,009 women that met the eligibility criteria. The mean age of the women was 39.9 years (Standard Deviation 9.01), and the vast majority of them were white women of reproductive age. Approximately 80% of the women were above 30 years old and 13.8% were postmenopausal. Most of the women (90%) had undergone cervical cytology screening in the past 5 years, and 77% of them were smokers. Only 4% of the women reported having started vaccination against HPV, which was anticipated since HPV vaccination was implemented in Greece in 2008 and was provided free of charge for women up to the age of 26. The complete demographic as well as clinical characteristics of the study population are shown in Table 1.

**Table 1:** Demographic and clinical characteristics of the study population.

	Eligible women (4009)
Age (years)	
*Mean(SD)*	39.88 (9.011)
*Median (range)*	40 (25–55)
Ethnic Origin	
*White*	4006
*Black*	3
Post menopausal	555 (13.8%)
HPV vaccinated	160 (4%)
Smoking history	
*Non smoker*	904 (22.55%)
*Present smoker*	3105 (77.45%)
Pap test in past 5 years	3605 (90%)
HPV test in past 5 years	200 (5%)
Colposcopy in past 5 years	361 (9%)
Study Cytology	
NILM	3785 (94.7%)
ASCUS or worse	211 (5.3%)
*ASCUS*	119 (3%)
*LSIL*	77 (1.9%)
*HSIL*	15 (0.4%)
*Pap result not available*	13 (0.3%)
HPV test results	
*Valid*	4006 (99.9%)
*Invalid*	3 (0.1%)

Of the 4009 women, 16 were excluded either because they did not have an LBC or an HPV test performed. Of the remaining 3,993 women, 604 were referred for colposcopy on the basis of an abnormal cytology (ASCUS or worse, N = 210) or because of 14HR HPV positive result with negative for intraepithelial lesions or malignancy (NILM) cytology (N = 394). However, due to various reasons (non-availability, immigration, unwillingness) colposcopy was not performed on 150 women, so the final analysis included 454 women who underwent colposcopy. In addition, 106 (2.6%) women with NILM cytology who tested HR-HPV negative were randomly assigned to undergo colposcopy to adjust for verification bias. In 93.4% (99/106) of these women the colposcopical findings were within normal limits [33]. For the remaining 7 women with abnormal colposcopic impression, the biopsies performed showed in only one case a low grade intraepithelial lesion (CIN1), (i.e.) no CIN2 or worse lesion was missed by either HPV DNA testing or cytology.

**Fig 2:**
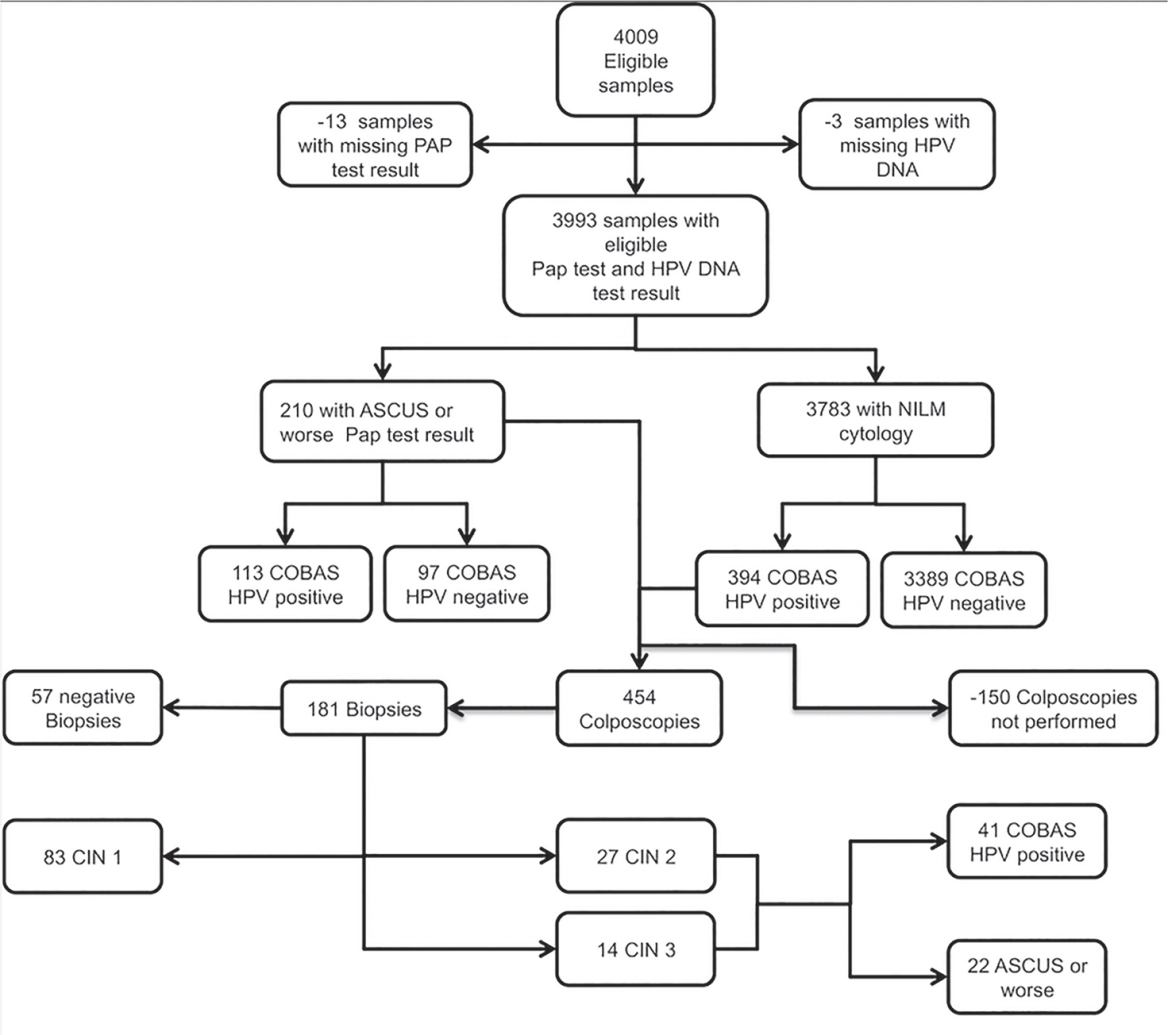
Flowchart for the liquid-based cytology and HPV DNA test performed on the study population.

The exclusions/non-attenders and the findings after the first screening round are given in the STARD [38] (Standards for Reporting of Diagnostic Accuracy) flowchart, which is shown in Fig. 2. Of the finally included 3,993 women who had both tests, the LBC results were NILM in 3,783 (94.7%), ASCUS in 118 (2.9%), LSIL in 77 (1.9%) and HSIL or ASC-H in 15 (0.4%). The HPV test results were positive in 507 women out of 3,993 (12.7%), of which 109 (2.7%) were positive for HPV 16, and 55 (1.4%) for HPV 18. There were 41 cases of CIN2 or worse finally diagnosed by histology (27 CIN2 and 14 CIN3). The prevalence of CIN2+ was 1.07%. The final diagnosis stratified by screening test result is given in Table 2.

**Table 2:** Final diagnosis stratified by LBC and HPV test results.

	LBC+	HPV+	LBC−/HPV−	LBC−/HPV+	LBC+/HPV−	LBC+/HPV+	Total
No Colposcopy*	69	98	3389	81	52	17	3539
Neg Colp / No biopsy	61	248	99**	212	25	36	273
Neg Biopsy	18	49	6**	39	8	10	57
CIN 1	40	71	1**	43	12	28	83
CIN 2	13	27	-	14	0	13	27
CIN 3	9	14	-	5	0	9	14
Total	210	507	3389	394	97	113	3993

LBC = Liquid based Cytology; HPV = Human Papilloma Virus; Neg Colp = Negative Colposcopy; Neg biopsy = Negative biopsy; CIN 1 or CIN2 or CIN3 = Cervical Intraepithelial Neoplasia grade 1 or 2 or 3.

* The row: “No Colposcopy” refers to women that did not have a colposcopy

** Cases concerning colposcopies performed for verification bias purposes are not added to the total.

Among the 3,783 women with NILM cytology the prevalence of 14HR HPV infection was 10.4% and the prevalence of HPV 16, 18 and the other 12HR HPV types was 1.3, 0.6 and 7.3% respectively, showing in all age groups a decreasing tendency with increasing age, as an indirect sign of transient HPV infection (Table 3). As far as the group of women with positive cytology (ASCUS or worse) is concerned, the HPV prevalence was 53.6, 36.2, 6.2 and 1.9% for any of the 14 high-risk types, for the 12 high-risk HPV types, for HPV 16 and HPV 18 respectively (Table 3). In this group of women a decrease in the prevalence of the respective HPV types is first noted after the age of 39 years—with the exception of HPV 18 -, an indirect sign of HPV infection persistency, as a probable cause of the abnormal cytology.

**Table 3:** Prevalence of HR-HPV in Women 25–55 years old with: NILM Cytology and ASCUS or worse Cytology.

		HPV Test Result							
Age Group	Total	14HR HPV(−)	14HR HPV(+)	12HR HPV(+)	HPV16(+)	HPV18(+)	HPV16(+)/HPV18(+)	HRHPV(+)/HPV16(+)	HRHPV(+)/HPV18(+)
***A*. *NILM NILM Cytology***									
25–29	625	77.3	22.7	14.9	3.5	1.4	0.2	1.9	0.6
30–39	1080	87.4	12.6	8.5	1.5	0.8	0.1	0.9	0.6
40–49	1388	94.0	6.0	4.5	0.8	0.2	-	0.1	0.3
50–55	690	95.2	4.8	4.3	-	0.3	-	0.1	-
Overall	3783	89.6	10.4	7.3	1.3	0.6	0.1	0.7	0.4
***ASCUS or worse Cytology***									
25–29	77	32.5	67.5	45.5	6.5	-	-	11.7	2.6
30–39	63	34.9	65.1	42.9	9.5	3.2	1.6	3.2	3.2
40–49	58	69.0	30.5	20.7	3.4	3.4	-	1.7	1.7
50–55	12	83.3	16.7	16.7	-	-	-	-	-
Overall	210	46.2	53.6	36.2	6.2	1.9	0.5	5.7	2.4

HPV = human papilloma virus; HR-HPV = high-risk HPV types; ASCUS = atypical squamous cells of undetermined significance; 14HR HPV(−) = HPV DNA test negative for the pooled 14 high-risk types; 14HR HPV(+) = HPV DNA test positive for the pooled 14 high-risk types; 12HR HPV(−) = HPV DNA test negative for the pooled 12 high-risk types except 16 and 18; 12HR HPV(+) = HPV DNA test positive for the pooled 12 high-risk types except 16 and 18; HPV16(+) = HPV DNA test positive for HPV 16; HPV18(+) = HPV DNA test positive for HPV 18; HPV16(+)/HPV18(+) = HPV DNA test positive for HPV16 and HPV18; HRHPV(+)/HPV16(+) = HPV DNA test positive for the pooled 12 high-risk types except 16 and 18 and for HPV16; HRHPV(+)/HPV18(+) = HPV DNA test positive for the pooled 12 high-risk types except 16 and 18 and for HPV18.

### Absolute risk of CIN2 or worse by HPV test result

Women positive for HPV DNA with normal cytology had an increased risk of harbouring CIN2 or worse. The overall estimated absolute risk for CIN 2 or worse in NILM women who were positive for HPV, ranged from 3.1 (95% CI 1.4–6.5) for 12 HR HPV positivity to 15.0 (95% CI 7.5–27.1) in women who were positive for HPV 16 (Table 4). These women as anticipated had the highest risk, followed by women positive for HPV 16 or 18 [13.8 (95% CI 7.6–23.2)] and women positive for HPV 18 only [12.9 (95% CI 4.2–30.8)]. The overall risk for 14 HR HPV positivity presented a slight increase, as women got older. This was also the case for the subgroup of HPV 16 or 18 positive women as well as for the 12 HR HPV positive women. Concerning HPV 16 only, the increase of the risk for the development of CIN2 or worse seen in women between 30–39 years old compared to women 25–29 was followed by a decrease for women 40 or older.

**Table 4:** Impact of HR-HPV on Estimated Absolute Risk of High-Grade Cervical Disease in Women With: NILM Cytology and ASCUS or worse Cytology.

	Age Group, years				
HPV Test Result	25–29	30–39	40–49	50–55	Overall
***CIN2 or worse***					
14HR HPV(+)	5.7 (2.3–12.4)	6.7 (3.3–13.7)	7.7 (2.9–17.8)	0.0 (0.0–15.5)	6.1 (3.8–9.5)
HPV16(+)	13.8 (4.5–32.6)	18.2 (6.0–41.0)	12.5 (0.7–53.3)	0.0 (0.0–94.5)	15.0 (7.5–27.1)
HPV18(+)	20.0 (3.5–55.8)	6.25 (0.3–32.3)	25.0 (1.32–78.1)	0.0 (0.0–94.5)	12.9 (4.2–30.8)
HPV16(+)/HPV18(+)	13.5 (5.1–29.6)	13.9 (5.2–30.1)	16.7 (2.9–49.1)	0.0 (0.0–80.2)	13.8 (7.6–23.2)
12HR HPV(+)	1.45 (0.1–8.9)	3.8 (1.0–11.5)	5.7 (1.5–16.6)	0.0 (0.0–16.6)	3.1 (1.4–6.5)
14HR HPV(−)	0.0 (0.0–1.0)	0.0 (0.0–0.5)	0.0 (0.0–0.4)	0.0 (0.0–0.7)	0.0 (0.0–0.1)
***CIN2 or worse***					
14HR HPV(+)	22.5 (11.4–38.9)	16.2 (6.8–32.7)	35.3 (15.3–61.4)	50.0 (2.7–97.3)	22.9 (15.2–32.8)
HPV16(+)	16.7 (2.9–49.1)	37.5 (10.2–74.1)	66.7 (12.5–98.2)	-*	30.4 (14.0–53.0)
HPV18(+)	66.7 (12.5–98.2)	20.0 (1.0–70.1)	66.7 (12.5–98.2)	-*	45.4 (18.1–75.4)
HPV16(+)/HPV18(+)	28.6 (9.6–58.0)	33.3 (11.3–64.6)	66.7 (24.1–94.0)	-*	37.5 (21.7–56.2)
12HR HPV(+)	19.2 (7.3–40.0)	8.0 (1.4–27.5)	18.2 (3.2–52.2)	50.0 (2.7–97.3)	15.6 (8.1–27.3)
14HR HPV(−)	0.0 (0.0–16.6)	0.0 (0.0–18.5)	0.0 (0.0–10.9)	0.0 (0.0–34.4)	0.0 (0.0–4.7)

HPV = human papilloma virus; HR-HPV = high-risk HPV types; ASCUS = atypical squamous cells of undetermined significance; 14HR HPV(−) = HPV DNA test negative for the pooled 14 high-risk types; 14HR HPV(+) = HPV DNA test positive for the pooled 14 high-risk types; 12HR HPV(−) = HPV DNA test negative for the pooled 12 high-risk types except 16 and 18; 12HR HPV(+) = HPV DNA test positive for the pooled 12 high-risk types except 16 and 18; HPV16(+) = HPV DNA test positive for HPV 16; HPV18(+) = HPV DNA test positive for HPV 18; HPV16(+)/HPV18(+) = HPV DNA test positive for HPV16 and HPV18; HRHPV(+)/HPV16(+) = HPV DNA test positive for the pooled 12 high-risk types except 16 and 18 and for HPV16; HRHPV(+)/HPV18(+) = HPV DNA test positive for the pooled 12 high-risk types except 16 and 18 and for HPV18

*No HPV positive cases with ASCUS+ cytology and CIN2+

Concerning women with abnormal cytology result a similar trend for the impact of HR HPV positivity on the risk of developing CIN2+ is observed. That is, the overall absolute risk for CIN2+ for women with ASCUS or worse cytology ranged from 15.6 (95% CI 8.1–27.3) to 45.4 (95% CI 18.1–75.4) for women positive for 12 HR HPV and for HPV 18 respectively. Women positive for HPV 16 showed a higher risk with increasing age (Table 4).

### Relative risk of CIN2 or worse by HPV test result

Positivity for a specific type of HPV plays an important role on the risk of developing cervical abnormalities. The relative risk of CIN 2 or worse by HPV status in women aged 25–29 years or ≥30 years with NILM as well as with abnormal (ASCUS or worse) cytology are provided in Table 5. The non-existence of false-negative 14HR HPV results makes the calculation of the relative risk of different HPV+ results compared to 14HR HPV negative results impossible. However, the relative risk for CIN 2 or worse was highest in cytologically normal women found to be HPV16 positive compared with HPV16 negative women in both age groups. In the group of women with NILM cytology, but also among women with abnormal cytology (ASCUS or worse), this risk was slightly greater in older compared to younger women [NILM: RR: 47.5 (95% CI 16.2–139.0) vs 32.7 (95% CI 6.2–172.7) and ASCUS or worse: 5.8 (95% CI 2.2–15.1) vs 1.2 (95% CI 0.3–5.1) respectively]. In contrast, the HPV18-related risk for CIN2+ between these two age groups of women was almost similar and, in addition, among young women with abnormal cytology it was greater than the HPV16-related [7.0 (95% CI 2.4–20.5) vs 1.2 (95% CI 0.3–5.1)].

**Table 5:** Relative Risk of High-Grade Cervical Disease in women 25–29 years old with NILM or ASCUS or worse cytology and in women 30–55 years old with NILM or ASCUS or worse cytology.

HPV Test Result	Relative Risk of CIN2+ (95% CI)
***NILM cytology (women 25–29 years old)***	
HPV16(+) vs HPV16(−)	32.7 (6.2–172.7)
HPV18(+) vs HPV18(−)	20.3 (4.0–102.6)
HPV16(+) vs 12HR HPV(+)	8.5 (0.8–89.1)
14HR HPV(+) vs 14HR HPV(−)	- *(no false negatives)*
***ASCUS or worse cytology (women 25–29 years old)***	
HPV16(+) vs HPV16(−)	1.2 (0.3–5.1)
HPV18(+) vs HPV18(−)	7.0 (2.4–20.5)
HPV16(+) vs 12HR-HPV (+)	- (no cases)
14HR-HPV(+) vs 14HR-HPV(−)	- (no false negatives)
***NILM cytology (women 30–55 years old)***	
HPV16(+) vs HPV16(−)	47.5 (16.2–139.0)
HPV18(+) vs HPV18(−)	21.1 (4.9–90.6)
HPV16(+) vs 12HR-HPV (+)	2.3 (0.5–10.7)
14HR-HPV(+) vs 14HR-HPV(−)	- *(no false negatives)*
***ASCUS or worse cytology (women 30–55 years old)***	
HPV16(+) vs HPV16(−)	5.8 (2.2–15.1)
HPV18(+) vs HPV18(−)	4.1 (1.4–12.4)
HPV16(+) vs 12HR-HPV (+)	4.1 (1.4–12.0)
14HR-HPV(+) vs 14HR-HPV(−)	- (no false negatives)

HPV = human papilloma virus; HR-HPV = high-risk HPV types; NILM = negative for intraepithelial lesion or malignancy; ASCUS = atypical squamous cells of undetermined significance; 14HR HPV(−) = HPV DNA test negative for the pooled 14 high-risk types; 14HR HPV(+) = HPV DNA test positive for the pooled 14 high-risk types; 12HR HPV(−) = HPV DNA test negative for the pooled 12 high-risk types except 16 and 18; 12HR HPV(+) = HPV DNA test positive for the pooled 12 high-risk types except 16 and 18; HPV16(+) = HPV DNA test positive for HPV 16; HPV18(+) = HPV DNA test positive for HPV 18; HPV16(+)/HPV18(+) = HPV DNA test positive for HPV16 and HPV18; HRHPV(+)/HPV16(+) = HPV DNA test positive for the pooled 12 high-risk types except 16 and 18 and for HPV16; HRHPV(+)/HPV18(+) = HPV DNA test positive for the pooled 12 high-risk types except 16 and 18 and for HPV18; CIN2 = Cervical Intraepithelial Neoplasia grade 2

### Test performance

The performance of every test or combination of tests was assessed by the calculation of sensitivity, specificity, positive and negative likelihood ratio, and positive and negative predictive value. This analysis was performed for the whole study population for the disease threshold CIN2+ or CIN3+ as well as for women 25–29 years and ≥30 years old separately for the disease threshold CIN2+ only.

All cases of CIN2+ were positive for cobas HPV test, therefore at the threshold of CIN2+ HPV testing had a sensitivity of 100% (95% CI 91.4%–100.0%), which was higher than the sensitivity of cytology [ASCUS+: 53.7% (95% CI 37.4%–69.3%)] to a statistically significant degree (*P*<0.05). The sensitivity difference (ASCUS+ cytology—14HR HPV testing) was -0.46 (95% CI: -0.61–-0.30). In contrast, the specificity of cytology [ASCUS or worse: 96.8% (95% CI 96.2%-97.4%), LSIL or worse: 98.8% (95% CI 98.4%–99.1%)] was significantly higher than the specificity of HPV testing [90.3% (95% CI 89.3%–91.2%)] (*P*<0.05). The specificity difference (ASCUS+ cytology—14HR HPV testing) was 0.07 (95% CI: 0.06–0.08). Testing for types 16&18 only had similar diagnostic accuracy indices to cytology. Positivity for the combination of the two tests (co-testing), that is positivity for either ASCUS+ or 14HR HPV testing, resulted in reduced specificity (Table 6).

**Table 6:** Accuracy indices for various tests and age groups of the study population.

Test	Sensitivity % (95% CI)	Specificity % (95% CI)	PLR (95% CI)	NLR (95% CI)	PPV % (95% CI)	NPV % (95% CI)
***Disease threshold CIN2+ for women 25–55 years old***						
**14HR HPV pos**.	100.0 (91.4–100.0)	90.3 (89.3–91.2)	10.3 (9.4–11.4)	0.0	10.0 (7.3–13.3)	100.0 (99.9–100.0)
**HPV 16/18 pos**.	58.5 (42.1–73.7)	97.5 (96.9–98.0)	23.4 (16.9–32.4)	0.4 (0.3–0.6)	20,17 (13.4–28.5)	99.5 (99.3–99.7)
**HPV 16 pos**.	39.0 (24.2–55.5)	98.2 (97.8–98.6)	22.1 (14.1–34.7)	0.6 (0.5–0.8)	19.3 (11.4–29.4)	99,33 (99.0–99.6)
**ASCUS or worse**	53.7 (37.4–69.3)	96.8 (96.2–97.4)	17,01 (12.2–23.8)	0.5 (0.3–0.7)	15.5 (10.0–22.5)	99.5 (99.2–99.7)
**LSIL or worse**	41.5 (26.3–57.9)	98.8 (98.4–99.1)	35.0 (22.0–55.8)	0.6 (0.5–0.8)	27.4 (16.8–40.2)	99.4 (99.1–99.5)
**Cotesting(either 14HR pos or ASCUS+)**	100.0 (91.4–100.0)	85.8 (84.6–86.8)	7.0 (6.5–7.6)	0.0	6.8 (4.9–9.1)	100 (99.9–100.0)
***Disease threshold CIN3+ for women 25–55 years old***						
**14HR HPV pos**.	100.0 (76.8–100.0)	89.7 (88.7–90.6)	9.7 (8.8–10.6)	0.0	3.4 (1.9–5.7)	100.0 (99.9–100.0)
**HPV16/18 pos**.	78.6 (49.2–95.3)	97.2 (96.6–97.7)	27.7 (20.0–38.7)	0.2 (0.1–0.6)	9.2 (4.4–15.1)	99.9 (99.8–100.0)
**HPV 16 pos**.	57.1 (28.9–82.3)	98.0 (97.5–98.5)	29.2 (17.6–48.4)	0.4 (0.2–0.8)	9.6 (4.2–18.1)	99.8 (99.6–99.9)
**ASCUS or worse**	64.3 (35.1–87.2)	96.5 (95.9–97.1)	18.6 (12.2–28.5)	0.4 (0.2–0.7)	6.4 (3.0–11.8)	99.9 (99.7–100.0)
**LSIL or worse**	57.1 (28.9–82.3)	98.6 (98.2–98.9)	40.5 (24.0–68.5)	0.4 (0.2–0.8)	12.9 (5.7–23.8)	99.8 (99.6–99.9)
**Cotesting(either 14HR pos or ASCUS+)**	100.0 (76.8–100.0)	85.2 (84.1–86.3)	6.7 (6.3–7.3)	0.0	2.3 (1.3–3.9)	100 (99.9–100.0)
***Disease threshold CIN2+ for women 25–29 years old***						
**14HRHPVpos**.	100.0 (78.2–100.0)	79.1 (75.7–82.2)	4.8 (4.1–5.6)	0.0	10.3 (5.9–16.4)	100.0 (99.3–100.0)
**HPV16/18 pos**.	53.3 (26.6–78.7)	93.6 (91.4–95.4)	8.4 (4.8–14.6)	0.5 (0.3–0.9)	16.7 (7.5–30.2)	98.8 (97.6–99.5)
**HPV 16 pos**.	40.0 (16.3–67.7)	94.4 (92.3–96.1)	7.2 (3.6–14.4)	0.6 (0.4–1.0)	14.6 (5.6–29.2)	98.5 (97.2–99.3)
**ASCUS or worse**	60.0 (32.3–83.7)	92.1 (89.6–94.2)	7.6 (4.6–12.6)	0.4 (0.2–0.8)	17.0 (8.1–29.8)	98.8 (97.5–99.6)
**LSIL or worse**	40.0 (16.3–67.7)	96.4 (94.5–97.8)	11.2 (5.3–23.8)	0.6 (0.4–0.9)	23.1 (9.0–43.6)	98.4 (96.9–99.2)
***Disease threshold CIN2+ for women ≥30 years old***						
**14HR HPV pos**.	100.0 (86.8–100.0)	92.5 (91.6–93.4)	13.4 (11.8–15.1)	0.0	9.9 (6.6–14.1)	100.0 (99.9–100.0)
**HPV 16/18 pos**.	61.5 (40.6–79.8)	98.3 (97.7–98.7)	35.5 (23.8–53.0)	0.4 (0.2–0.6)	22.5 (13.5–34.0)	99.7 (99.4–99.8)
**HPV 16 pos**.	38.5 (20.2–59.4)	99.0 (98.6–99.3)	38.1 (21.0–69.2)	0.6 (0.5–0.8)	23.8 (12.0–39.4)	99.5 (99.2–99.7)
**ASCUS or worse**	50,0 (29.9–70.1)	97.7 (97.1–98.1)	21.3 (13.7–33.3)	0.5 (0.3–0.7)	14.6 (8.0–23.7)	99.6 (99.3–99.8)
**LSIL or worse**	42.3 (23.3–63.1)	99.2 (98.9–99.5)	54.9 (30.3–99.5)	0.6 (0.42–0.8)	30.6 (16.3–48.1)	99.5 (99.4–99.7)

CIN2+ = cervical intraepithelial neoplasia grade 2 or worse histology; PLR = Positive Likelihood Ratio; NLR = Negative Likelihood Ratio; PPV = Positive Predictive Value; NPV = Negative Predictive Value; HPV = human papilloma virus; 14HR HPV pos. = HPV DNA test positive for the pooled 14 high-risk types; HPV 16/18 pos. = HPV DNA test positive for HPV 16 or HPV 18 or both; HPV 16 pos. = HPV DNA test positive for HPV 16; ASCUS or worse = atypical squamous cells of undetermined significance or worse cytology; LSIL or worse = low grade squamous intraepithelial lesion or worse cytology

At the threshold of CIN3+ HPV testing had a sensitivity of 100% (95% CI 76.8%–100.0%), which was higher than the sensitivity of cytology [ASCUS+: 64.3% (95% CI 35.1%–87.2%)] but the difference did not reach statistical significance. The sensitivity difference (ASCUS+ cytology—14HR HPV testing) was -0.36 (95% CI: -0.61–-0.07). In contrast, the specificity of cytology [ASCUS+: 96.5% (95% CI 95.9%–97.1%), LSIL+: 98.6% (95% CI 98.2%–98.9%)] was significantly higher than the specificity of HPV testing [89.7% (95% CI 88.7%–90.6%)] (*P*<0.05). The specificity difference (ASCUS+ cytology—14HR HPV testing) was 0.07 (95% CI: 0.06–0.08). Testing for type 16 only had similar diagnostic accuracy indices to cytology. Testing for types 16&18 only had at least equal accuracy compared to cytology for CIN3+ but not in a statistically significant degree. The combination of the two tests (co-testing) resulted again in reduced specificity (Table 6).

With the exception of 14HR HPV testing, which has a sensitivity of 100% in both cases, changing the threshold from CIN2+ to CIN3+ has led to an increase in sensitivity and NPV for all tests examined. The largest increase in sensitivity, from 58.5% (95% CI 42.1%–73.7%) to 78.6% (95% CI 49.2%–95.3%), was noted for HPV 16 or 18 positivity. PLR was slightly increased if the threshold was CIN3+ for all tests apart from positivity for the 14HR HPV types, and NLR as well as PPV markedly decreased. Comparison of the diagnostic accuracy of the cobas HPV test to cytology with the McNemar’s test revealed a statistically significant difference (*P*<0.0001).

The addition of HPV testing resulted in 188% more colposcopy referrals than cytology alone (210 cases with ASCUS+ vs 604 cases with ASCUS+ and/or 14HR HPV positive), but also in 86% more cases of CIN2+ detected (22 cases with ASCUS+ vs 41 cases with ASCUS+ and/or 14HR HPV positive). The number of colposcopies needed to detect a CIN2+ after a positive cytology or positive 14HR HPV testing or both (co-testing) was 11.1 [41 CIN2+ detected after performance of 454 colposcopies (454/41)], whereas the respective number for 14HR HPV testing alone was 10.0 (409/41) and for cytology (ASCUS+) alone was 6.4 (141/22).

HPV infection in general, and especially asymptomatic or transient HPV infections are more common in younger age groups compared to older, whereas the opposite is the case for the prevalence of high-grade cervical lesions (CIN2 or worse) [39,40]. Therefore, two separate analyses were performed concerning test accuracy indices for the detection of CIN2 or worse lesions. The first analysis involved women from 25 to 29 and the second women 30 to 55 years old (Table 6). In general, sensitivity of either HPV DNA testing or cytology does not show a statistically significant change between younger and older women, whereas the opposite is observed for specificity, which increased for all tests performed in older women. The specificity of the cobas HPV test was significantly higher in the group of women ≥30 years compared to younger women [92.5% (95% CI 91.6%–93.4%) vs 79.1% (95% CI 75.7%–82.2%)] but remained significantly lower than cytology [97.7% (95% CI 97.1%–98.1%)]. As expected, a statistically significant increase in PLR was noted in older women concerning all tests. It is noteworthy that regarding PLR, the slight superiority of HPV 16/18 detection compared to positive cytology (ASCUS+) becomes more marked in older women. Changing the onset of screening from 25 to 30 years of age, does not influence sensitivity of the various tests, however it is interesting that both, the specificity and the PLR of HPV testing (14HR HPV) are significantly increased [90.3% (95% CI 89.3%–91.2%) vs 92.5% (95% CI 91.6%-93.4%) and 10.3 (95% CI 9.4–11.4) vs 13.4 (95% CI 11.8–15.1) respectively]. Concerning cytology an increase of these two accuracy indices was also noted, however not to a statistically significant degree.

Finally, Table 7 summarises sensitivity, PPV, RR and LR for various combinations of HPV 16/18 genotyping and LBC as methods of triage for colposcopy of HPV-positive women for the detection of CIN2 or worse. The sensitivity of cytology alone (ASCUS or worse) as a method of triage of all HPV-positive women was 53.7% (95% CI 37.4%–69.3%) and the PLR was 11.2 (95% CI 8.2–15.4).

**Table 7:** Performance of various tests combinations for triage to colposcopy of HPV positive women for the detection of CIN2+.

	Sensitivity	PPV	RR	PLR
CIN2 or worse	% (95%CI)	% (95% CI)	(95%CI)	(95%CI)
None (all HPV+ to colposcopy)	100.0 (91.4–100.0)	10.0 (7.3–13.3)	-	-
ASCUS or worse	53.7 (37.4–69.3)	10.4 (6.3–14.6)	20.8 (11.4–37.8)	11.2 (8.2–15.4)
LSIL or worse	41.5 (26.3–57.9)	18.5 (10.5–26.4)	30.1 (16.7–54.0)	21.9 (14.3–33.5)
HSIL or worse	17.1 (5.6–25.6)	46.7 (21.4–71.9)	54.6 (28.9–103.2)	80.5 (30.6–211.68)
HPV16+	39.0 (24.2–55.4)	14.8 (8.1–21.5)	23.1 (12.7–42.0)	16.8 (10.9–25.9)
HPV16+ or ASCUS or worse	75.6 (65.4–88.8)	10.8 (7.2–14.4)	40.2 (19.9–81.1)	11.7 (9.5–14.5)
HPV16+ and ASCUS or worse	17.1 (5.6–28.6)	25.0 (9.0–41.0)	29.3 (14.2–60.3)	32.3 (14.5–71.6)
HPV16+ or LSIL or worse	65.9 (51.3–80.4)	15.1 (9.9–20.4)	41.5 (22.1–77.7)	17.3 (13.2–22.7)
HPV16+ and LSIL or worse	14.3 (3.8–25.5)	31.6 (10.7–52.5)	36.0 (17.2–75.4)	44.7 (17.8–111.7)
HPV16+ or HSIL or worse	53.7 (38.4–68.9)	18.8 (11.7–25.9)	38.5 (21.4–69.2)	22.4 (15.8–31.7)
HPV16+ and HSIL or worse	-	-	-	-
HPV16+ or HPV18+	58.5 (42.1–73.7)	15.4 (9.7–21.0)	34.9 (19.1–63.5)	17.6 (12.9–23.9)
(HPV16+ or HPV18+) or ASCUS or worse	92.6 (91.8–93.5)	10.4 (7.1–13.7)	54.9 (24.5–122.8)	11.3 (9.4–13.5)
(HPV16+ or HPV18+) and ASCUS or worse	29.3 (15.3–43.2)	32.4 (17.3–47.5)	44.4 (24.6–80.1)	46.4 (25.1–85.9)
(HPV16+ or HPV18+) or LSIL or worse	78.0 (65.4–90.7)	14.5 (9.8–19.1)	60.9 (29.5–126.4)	16.4 (13.2–20.3)
(HPV16+ or HPV18+) and LSIL or worse	22.0 (9.3–34.6)	39.1 (19.2–59.1)	48.7 (26.3–90.2)	62.3 (28.6–135.7)
(HPV16+ or HPV18+) or HSIL or worse	68.3 (54.0–82.5)	17.4 (11.5–23.2)	51.5 (27.2–97.5)	20.4 (15.6–26.6)
(HPV16+ or HPV18+) and HSIL or worse	7.3 (0.0–15.3)	50.0 (10.0–90.0)	52.7 (22.3–124.5)	96.8 (20.1–465.3)

CIN2+ = cervical intraepithelial neoplasia grade 2 or worse histology; PPV = Positive Predictive Value; RR = Relative Risk; PLR = Positive Likelihood Ratio; HPV = human papilloma virus; ASCUS or worse = atypical squamous cells of undetermined significance or worse cytology; LSIL or worse = low grade squamous intraepithelial lesion or worse cytology; HSIL or worse = high grade squamous intraepithelial lesion or worse cytology; HPV 16+ = HPV DNA test positive for HPV 16; HPV 16+ or HPV 18+ = HPV DNA test positive for HPV 16 or HPV 18 or both.

By combining genotyping with cytology, testing positive for HPV16 or ASCUS+ cytology, as well as testing positive for HPV16 or HPV18 or ASCUS+ cytology had a markedly higher sensitivity of 75.6% (95% CI 65.4%–88.8%) and 92.6% (95% CI 91.8%–93.5%) respectively than ASCUS+ alone, the former being marginally significant and the latter being clearly significant and the highest among all test combinations. By replacing ASCUS+ by LSIL+ or HSIL+, combined with HPV16 testing alone or HPV 16 or HPV 18 or both, a gradual decrease in sensitivity was noted, although all rates were not statistically significantly different than ASCUS or worse cytology alone. Testing positive for HPV 16 or HPV 18 or both, and additionally ASCUS+ cytology would significantly increase PPV and PLR for the histological identification of CIN2+, in comparison to ASCUS+ alone [32.4% (95% CI 17.3%–47.5%) and 46.4 (95% CI 25.1–85.9) vs 10.4% (95% CI 6.3%–14.6%) and 11.2 (95% CI 8.2–15.4) respectively]. Similar significant increase in PPV and PLR in HPV 16/18 cases is noted when ASCUS+ is replaced by LSIL+ compared to ASCUS+ cytology alone [39.1% (95% CI 19.2%–59.1%) and 62.3 (95% CI 28.6–135.7) vs 10.4% (95% CI 6.3%–14.6%) and 11.2 (95% CI 8.2–15.4) respectively].

Detection of HSIL or worse cytology or HPV 16/18 genotyping for the triage of HPV positive women has a slight, not statistically significant increase of sensitivity and PPV than ASCUS or worse cytology alone [68.3% (95% CI 54.0%–82.5%) vs 53.7% (95% CI 37.4%–69.3%) and 17.4% (95% CI 11.5%–23.2%) vs 10.4% (95% CI 6.3%–14.6%)]. However, considering that the PPV of HSIL alone is 46.7% (95% CI 21.4%–71.9%), testing positive also for HPV 16 or 18 or both would result in an almost similar PPV of 50.0% (95% CI 10.0%–90.0%), whereas the sensitivity would drop from 17.1% (95% CI 5.6%–25.6%) to 7.3% (95% CI 0.0%–15.3%)]. According to that, the addition of HPV 16/18 genotyping to HSIL cytology would not improve diagnostic accuracy concerning triaging HPV positive women.

Finally, no statistically significant changes in relative risk between the different combinations of triage tests used to detect CIN2 or worse cases among HPV-positive women were noted.

## Discussion

Many things have changed since 1943 when Papanicolaou and Traut published their paper on the role of the vaginal smear in cervical cancer diagnosis [41], leading ultimately to the implementation of cytology in cervical cancer screening programs worldwide [42] and subsequently to the reduction of the incidence and mortality due to the disease. However, it seems that the effect of cytology on cervical cancer epidemiology has reached a limit and cannot further easily improve the burden of cervical cancer worldwide. It is worth mentioning that the overall performance of cytology for the detection of CIN2+ cervical lesions in a review of studies from Europe and North America was poor since it presented sensitivity of 53% [43] (in our study the sensitivity of cytology alone was 53.7%). Several attempts to improve cytology-based screening have been made including the modification of cytological terminology [32], and the implementation of novel techniques such as liquid-based cytology. The latter has not proven to be statistically significantly better than conventional cytology in terms of sensitivity and specificity [44], but it has offered a new medium for the cervical cells and hence new possibilities for their molecular examination in the era of HPV DNA testing.

Since the discovery of the role of HPV on cervical cancer [5,6] many tests have been developed to identify HPV infection, and tested by screening patient groups. It has been shown, that HPV test results predict the risk of cervical cancer and its precursors better and sooner than cytological abnormalities [23,45]. Thus, apart from the use of HPV DNA testing for triage of borderline cytology, as well as for follow-up after surgical treatment of CIN, HPV DNA testing has been proved as accurate methodology for primary cervical cancer screening, either in conjunction with cytology, as a co-test [46], or as a stand-alone test [21,35,43,47–50]. Ronco *et al*. taking into account the follow-up data of 176,464 women from 4 European studies have shown that HPV DNA-based screening alone provides a 60–70% better protection against cervical cancer compared to cytology [15]. Very recently, Gage *et al*. used data from over one million women aged 30 to 64 years and screened for cervical cancer at Kaiser Permanente Northern California since 2003 and found that, overall, the three-year risk of invasive cervical cancer following a HPV-negative result was much lower than the 3-year risk following a cytology-negative result (11 vs 20, P < 0.0001) and lower than the 5-year risk following a HPV-negative/cytology-negative co-test (11 vs 14, P = 0.21) [51]. Furthermore, in the post HPV-vaccination era, the expected decrease in the prevalence of cervical lesions could lead to a decrease in the performance of cytology as a method of primary cervical cancer screening [52]. However, in the present study this could not be evident since the percentage of women vaccinated against HPV was only 4% and HPV vaccination in Greece started at 2008.

On April 2014 the US Food and Drug Administration (FDA) approved the cobas HPV test by Roche Molecular Systems, Incorporated, Pleasanton, California (http://www.fda.gov/newsevents/newsroom/pressannouncements/ucm394773.htm) as a stand- alone method for cervical cancer screening. Cobas HPV test is a PCR based HPV DNA test which is among those that have satisfactory performance in terms of sensitivity and specificity for the detection of high grade cervical lesions [53]. This test has been validated initially by the research group of Meijer [54] and then by the large ATHENA study [10–13,55], the results of which have shown that its performance was comparable to the Hybrid Capture 2 HPV test (QIAGEN, Gaithersburg, MD) [10]. The latter is an established standard worldwide, and served as a comparator in the guidelines for accurate HPV testing methods [55,56].

The objective of the present study is to compare the performance of HPV DNA testing, using the cobas HPV test as stand-alone cervical cancer screening method, to that of liquid-based cytology. In the present article it is shown that the cobas HPV test has superior sensitivity to cytology (ASCUS or worse) in the detection of CIN2+ and CIN3+ (cytology alone had a sensitivity of 53.7% and 64.3% respectively), whereas HPV testing using the cobas system presented a sensitivity of 100% for both histological thresholds—the absolute 100% is due probably to the fact that the sample tested was relatively small-. This makes the cobas HPV test a more suitable primary screening test, in comparison to cytology, as it would reduce the false negative results, which is the main weakness of cytology-based screening programs as it has been shown in cervical cancer audits. In addition, this study questions the benefit of the simultaneous use of cytology with HPV testing, as there were no cases missed by HPV testing that were picked up by cytology. At the same time the NPV of 14 HR HPV testing both, for the threshold of CIN2+ and CIN3+, as well as for women aged 25–29 years and for women ≥30 years, was 100%, so it could not be further increased by the use of cytology. Thus, according to the results of the present study, and in accordance with the results of the mentioned large studies in Europe [15,35,47,48,50] and in the USA [51,55,57] HPV testing could be used as a stand-alone primary test for cervical cancer screening.

The increased sensitivity of the cobas HPV test is achieved at the cost of a decreased specificity compared to cytology (ASCUS or worse), and an increased referral rate for colposcopy since 10 colposcopies needed to be performed in order to diagnose one case of CIN 2+ compared to 6.4 in case of cytology. However, HPV testing alone has a smaller referral rate for colposcopy than co-testing (10:11) while offering equal outcome. It is possible that the rather high percentage of HR HPV positive women in the presented study (12.7% of all women and 10.4% of all NILM women) could have contributed to the rather low specificity of the cobas HPV test used (90.3%). In this regard, it is noteworthy that in the American ATHENA trial, where the overall HR HPV prevalence was 10% and the percentage of HR HPV positive NILM population was 8%, the adjusted specificity of HPV testing was 90.2%, similar to the reported herein [11].

The sub-analysis performed for women aged below and above 30 revealed that specificity of the cobas HPV test for the detection of CIN2+ was 79.1% (95% CI 75.7%–82.2%) for women between 25 and 29 years old and significantly lower compared to women above 30, for whom the specificity of the test reached 92.5% (95% CI 91.6%–93.4%). It is also noteworthy, that changing the starting age of screening from 25 to 30 years, leads to significant increase on both, specificity and PLR of HPV testing. Therefore, using the test as a method of primary screening only in women 30 or older could drop the referral rate.

It is obvious, that women with transient HPV infection or with cervical lesions that do not need treatment at the time of initial evaluation should be distinguished from women with precancerous or invasive lesions, and in this regard, triaging of HPV-positive women for colposcopy, in order to identify the women at risk, is inevitable. Regarding the time schedule of this approach, it is not clear if a re-evaluation with HPV testing should be performed in 1 or 2 year interval in order to identify the persistent HPV infections [58], or if an immediate triage strategy is appropriate [45,59,60].

Cytological triage of a positive HPV test has been primarily proposed as the ideal method of identifying cellular abnormalities in HPV-infected cervices, and consequently as an appropriate method of reducing colposcopy referrals [35,58]. However, the realization of the different long-term transforming potentials of the various oncogenic HPV types [24,30] resulted in routine use of molecular tests assessing the presence or absence of the genome of these genotypes, and in particular of HPV 16 and HPV 18, as these types have the highest oncogenic potential, in order to triage HPV-positive women. According to the large ATHENA trial, it has been shown that HPV 16/18 genotyping alone has similar sensitivity to ASCUS or worse cytology alone, and, in addition, the combination of the two tests has a better sensitivity than that of a cytology alone test with a threshold of ASCUS or worse, leading to the conclusion that HPV-based screening strategies with HPV 16/18 genotyping could provide a more efficient modality for cervical cancer screening than cytology-based methods [11]. Based on the results of our study (in which all CIN2+ cases tested HPV positive) genotyping for HPV16, HPV 18, or both alone had similar or even better diagnostic accuracy indices compared to LBC (ASCUS or worse) alone for triage to colposcopy of women aged 25 or older who tested positive for HPV [sensitivity: 58.5% (95% CI 42.1%–73.7%) vs 53.7% (95% CI 37.4%–69.3%) and specificity: 97.5% (95% CI 96.9%–98.0%) vs 96.8% (95% CI 96.2%–97.4%) for identification of CIN2+; sensitivity: 78.6% (95% CI 49.2%–95.3%) vs 64.3% (95% CI 35.1%–87.2%) and specificity: 97.2% (95% CI 96.6%–97.7%) vs 96.5% (95% CI 95.9%–97.1%) for identification of CIN3+]. Sensitivity as well as specificity of HPV 16/18 genotyping alone compared to cytology (ASCUS+) alone for the detection of CIN2+ between the two age groups of women (25–29 years and ≥30 years) do not differ significantly. However, it should be emphasized that specificity rates increase significantly for both tests in women 30 years or older [HPV 16/18 genotyping: 93.6% (95% CI 91.4%–95.4%) vs 98.3% (95% CI 97.7%–98.7%) and ASCUS or worse cytology: 92.1% (95% CI 89.6%–94.2%) vs 97.7% (95% CI 97.1%–98.1%) for age groups 25–29 years and ≥30 years respectively]. In fact, both age groups demonstrated higher specificity for HPV genotyping compared to cytology, but not in a statistically significant degree. Thus, genotyping for these two particular high-risk HPV types, which has been shown to be associated with the majority (~70%) of invasive cervical cancers [25,61,62], could replace cytology in this triage process, both, in young women (aged 25–29 years), and even better in older women (aged ≥30 years). In the case of the cobas HPV test, this is particularly convenient, because this system for HPV DNA testing allows for the individual genotyping of type 16 as well as of type 18 simultaneously to the detection of the 14-pooled high-risk HPV genotypes.

On the other hand, by using genotyping for HPV 16, or HPV 18, or both as an additional or complementary triage strategy to ASCUS+ cytology in order to detect CIN2 or worse histology in HPV-positive women, it has been shown that the combination of the two tests can markedly improve sensitivity for the detection of CIN2+ [from 53.7% (ASCUS+ cytology alone) to 75.6% (ASCUS+ cytology and HPV 16 genotyping) and to 92.6% (ASCUS+ cytology and HPV 16/18 genotyping)]. Sensitivity rates follow similar trend when ASCUS or worse is replaced by LSIL or worse cytology. Testing positive for HPV 16 or HPV 18 or both and additionally ASCUS+ cytology would also significantly increase PPV and PLR for the identification of CIN2+, in comparison to ASCUS+ alone [32.4% and 46.4 vs 10.4% and 11.2 respectively]. Regarding cytology of HSIL or worse, the addition of HPV 16/18 genotyping would result in reduced sensitivity and similar PPV, a sign for the relatively low additive value of 16/18 genotyping in HPV-positive women if a HSIL+ cytology result is present.

As a conclusion, triaging of HR HPV positive women with HPV 16/18 genotyping alone has a similar or slightly better performance than using LBC alone, whereas combining cytological indices with HPV 16/18 genotyping results in an even more sensitive method to triage HPV-positive women than either genotyping or cytology alone. The latter, however, can only be achieved at the cost of performing two different diagnostic procedures, (i.e.) HPV 16/18 genotyping and LBC, additionally to the initial HPV testing. On the contrary, genotyping for HPV 16/18 using the cobas HPV test as alternative to cytology for triaging HPV-positive women, can be performed by one sole diagnostic procedure offering concurrently HPV testing and HPV 16/18 genotyping results.

The superior sensitivity of HPV testing is an established finding in the literature, but it has also been shown that there are variations in performance depending on the HPV testing method used and the study population especially between continents [31]. Therefore it is essential to study a particular and clinically validated test as the cobas HPV test in a particular population prior to the proposal on HPV testing inclusion in the screening program of the particular country. The present study aims to test the performance of cervical cytology and HPV DNA testing with 16/18 genotyping on a relatively small sample of about 4000 women residing in Greece. This sample is not quite representative of the Greek female population since it consists mostly of women living in urban areas. The centres participating in the study are referral hospitals carrying out also cervical cancer screening for the local population. However, the distribution of the participating centres across the country is more or less acceptable in terms of representation of the Greek mainland. This partly explains the relatively high HPV infection prevalence among the study population that reached 12.7%. The same figure drawn from a study on HPV prevalence using a more representative sample of the Greek female population, tested mainly by Hybrid Capture 2, was 5.8% [63,64], concordant to HPV prevalence reported for Southern Europe [5.7% [65]; 8.8% [66]], whereas the respective prevalence in the US reported by Castle *et al*. using cobas was 10% [11]. It is obvious, however, that the use of different methods for the detection of HPV DNA could play a role in the differences noted concerning HPV prevalence.

The design of our study, in some aspects and in a much smaller scale, is similar to that of the larger ATHENA trial and, based on the respective results, we drew similar conclusions. As weaknesses of our study one can notice the relatively small sample of women tested, as well as the fact that it is more subject to verification bias since double screen negatives were not offered colposcopy, apart from a small fraction of the NILM and HPV-negative women used for verification bias adjustment. Taking in mind that this percentage of double negative women tested was relatively small, there were no cases of CIN2+ found in the random sample of double negative women that had colposcopy, making actual adjustments of the results not necessary. This could cause an overestimation of the HPV test sensitivity but should not affect the relative difference between HPV testing and cytology. Apart from that, the lack of histological verification in all HPV-positive or/and cytology-positive women by using the presence of abnormal colposcopic impression as a prerequisite for the performance of biopsies, as well as the relatively high number of HPV-positive or/and cytology-positive women lost to colposcopy, represent additional limitations in our study, which could cause a diminished identification of women with high-grade disease. This fact could probably affect to some extent the final accuracy of the two screening tests.

As strengths of our study one could note the use of LBC, the fact that all women enrolled had both, HPV DNA testing and cytology performed, that cytology examinations were performed at the corresponding cytology laboratories of the hospitals cooperating in the study reflecting real-life routine laboratory conditions, that cytologists weren’t aware of the HPV DNA test results, nor were molecular biologists of the cytology results, that colposcopy and histology were conducted in each of the cooperating hospitals by experienced specialists and pathologists were not aware of the HPV DNA test result. As an additional strength of the present study one could consider the sub-analysis performed for women aged below and above 30, in order to identify the best option for starting age of cervical cancer screening, by assessing the performance of HPV DNA testing with genotyping as well as of LBC for the two age groups.

In summary, our study is the first multicenter trial in Greece evaluating the performance of HR HPV DNA testing with concurrent HPV 16/18 genotyping in comparison to LBC for primary cervical cancer screening. According to our results from the first screening round, HPV DNA testing has much better sensitivity than cytology to identify high-grade cervical lesions (CIN2+ or CIN3+), with slightly lower specificity. Changing the starting age of screening from 25 to 30 years, leads to significant increase on both, specificity and PLR of HPV DNA testing, advocating for its optimal use in women over 30 years of age. Additionally, genotyping for HPV 16 or HPV 18 or both in order to detect high-grade lesions has similar sensitivity and specificity to ASCUS or worse cytology in young (25–29 years) as well as in older women (30–55 years). In conclusion, HPV DNA testing with concurrent individual genotyping for HPV 16, HPV 18 or both, could represent a more accurate methodology for primary cervical cancer screening in comparison to liquid-based cytology.

## References

[1] Quinn M, Babb P, Jones J, Allen E. Effect of screening on incidence of and mortality from cancer of cervix in England: evaluation based on routinely collected statistics. BMJ. 1999;318: 904–908. 10102852 10.1136/bmj.318.7188.904 PMC27810

[2] Jemal A, Ward E, Thun M. Declining death rates reflect progress against cancer. PLoS One. 2010;5: e9584 10.1371/journal.pone.0009584 20231893 PMC2834749

[3] Koss LG The Papanicolaou test for cervical cancer detection. A triumph and a tragedy. JAMA. 1989;261: 737–743. 2642983

[4] Bosch FX, Munoz N, Shah KV, Meheus A. Second International Workshop on the Epidemiology of Cervical Cancer and Human Papillomaviruses. Int J Cancer. 1992;52: 171–173. 1325948 10.1002/ijc.2910520202

[5] zur Hausen H. Human papillomaviruses and their possible role in squamous cell carcinomas. Curr Top Microbiol Immunol. 1977;78: 1–30. 202434 10.1007/978-3-642-66800-5_1

[6] Walboomers JM, Jacobs MV, Manos MM, Bosch FX, Kummer JA, Shah KV et al Human papillomavirus is a necessary cause of invasive cervical cancer worldwide. The Journal of pathology. 1999;189: 12–19. 10451482 10.1002/(SICI)1096-9896(199909)189:1<12::AID-PATH431>3.0.CO;2-F

[7] Bosch FX, de Sanjose S. Chapter 1: Human papillomavirus and cervical cancer—burden and assessment of causality. J Natl Cancer Inst. 2003;Monogr: 3–13. 10.1093/oxfordjournals.jncimonographs.a003479 12807939

[8] Bouvard V, Baan R, Straif K, Grosse Y, Secretan B, El Ghissassi F. et al A review of human carcinogens—Part B: biological agents. Lancet Oncol. 2009;10: 321–322. 19350698 10.1016/s1470-2045(09)70096-8

[9] Bulkmans NW, Rozendaal L, Snijders PJ, Voorhorst FJ, Boeke AJ, Zandwijken GR, et al POBASCAM, a population-based randomized controlled trial for implementation of high-risk HPV testing in cervical screening: design, methods and baseline data of 44,102 women. Int J Cancer. 2004;110: 94–101. 15054873 10.1002/ijc.20076

[10] Stoler MH, Wright TC, Sharma A, Apple R, Gutekunst K, Wright TL, et al High-Risk Human Papillomavirus Testing in Women With ASC-US Cytology: Results From the ATHENA HPV Study. American Journal of Clinical Pathology. 2011;135: 468–475. 10.1309/AJCPZ5JY6FCVNMOT 21350104

[11] Castle PE, Stoler MH, Wright TC Jr, Sharma A, Wright TL, Behrens CM. Performance of carcinogenic human papillomavirus (HPV) testing and HPV16 or HPV18 genotyping for cervical cancer screening of women aged 25 years and older: a subanalysis of the ATHENA study. Lancet Oncol. 2011;12: 880–890. 10.1016/S1470-2045(11)70188-7 21865084

[12] Wright TC Jr, Stoler MH, Sharma A, Zhang G, Behrens C, Wright TL, et al Evaluation of HPV-16 and HPV-18 genotyping for the triage of women with high-risk HPV+ cytology-negative results. Am J Clin Pathol. 2011;136: 578–586. 10.1309/AJCPTUS5EXAS6DKZ 21917680

[13] Wright TC Jr, Stoler MH, Behrens CM, Apple R, Derion T, Wright TL. The ATHENA human papillomavirus study: design, methods, and baseline results. Am J Obstet Gynecol. 2012;206: 46 e41–46 e11. 10.1016/j.ajog.2011.07.024 21944226

[14] Dillner J. Primary human papillomavirus testing in organized cervical screening. Curr Opin Obstet Gynecol. 2013;25: 11–16. 10.1097/GCO.0b013e32835c5d10 23299089

[15] Ronco G, Dillner J, Elfstrom KM, Tunesi S, Snijders PJ, Arbyn M, et al Efficacy of HPV-based screening for prevention of invasive cervical cancer: follow-up of four European randomised controlled trials. Lancet. 2014;383: 524–532. 10.1016/S0140-6736(13)62218-7 24192252

[16] Arbyn M, Buntinx F, Van Ranst M, Paraskevaidis E, Martin-Hirsch P, Dillner J. Virologic versus cytologic triage of women with equivocal Pap smears: a meta-analysis of the accuracy to detect high-grade intraepithelial neoplasia. J Natl Cancer Inst. 2004;96: 280–293. 14970277 10.1093/jnci/djh037

[17] Agorastos T, Dinas K, Lloveras B, de Sanjose S, Kornegay JR, Bonti H, et al Human papillomavirus testing for primary screening in women at low risk of developing cervical cancer. The Greek experience. Gynecologic oncology. 2005;96: 714–720. 15721416 10.1016/j.ygyno.2004.11.042

[18] Mayrand MH, Duarte-Franco E, Rodrigues I, Walter SD, Hanley J, Ferenczy A, et al Human papillomavirus DNA versus Papanicolaou screening tests for cervical cancer. N Engl J Med. 2007;357: 1579–1588. 17942871 10.1056/NEJMoa071430

[19] Leinonen M, Nieminen P, Kotaniemi-Talonen L, Malila N, Tarkkanen J, Laurila P, et al Age-specific evaluation of primary human papillomavirus screening vs conventional cytology in a randomized setting. J Natl Cancer Inst. 2009;101: 1612–1623. 10.1093/jnci/djp367 19903804

[20] Cuzick J, Clavel C, Petry KU, Meijer CJ, Hoyer H, Ratnam S, et al Overview of the European and North American studies on HPV testing in primary cervical cancer screening. Int J Cancer. 2006;119: 1095–1101. 16586444 10.1002/ijc.21955

[21] Sankaranarayanan R, Nene BM, Shastri SS, Jayant K, Muwonge R, Budukh AM, et al HPV screening for cervical cancer in rural India. N Engl J Med. 2009;360: 1385–1394. 10.1056/NEJMoa0808516 19339719

[22] Cuzick J, Szarewski A, Mesher D, Cadman L, Austin J, Perryman K, et al Long-term follow-up of cervical abnormalities among women screened by HPV testing and cytology-Results from the Hammersmith study. Int J Cancer. 2008;122: 2294–2300. 10.1002/ijc.23339 18240149

[23] Elfstrom KM, Smelov V, Johansson AL, Eklund C, Naucler P, Arnheim-Dahlstrom L, et al Long term duration of protective effect for HPV negative women: follow-up of primary HPV screening randomised controlled trial. BMJ. 2014;348: g130 10.1136/bmj.g130 24435414 PMC3898575

[24] Khan MJ, Castle PE, Lorincz AT, Wacholder S, Sherman M, Scott DR, et al The elevated 10-year risk of cervical precancer and cancer in women with human papillomavirus (HPV) type 16 or 18 and the possible utility of type-specific HPV testing in clinical practice. J Natl Cancer Inst. 2005;97: 1072–1079. 16030305 10.1093/jnci/dji187

[25] de Sanjose S, Quint WG, Alemany L, Geraets DT, Klaustermeier JE, Lloveras B, et al Human papillomavirus genotype attribution in invasive cervical cancer: a retrospective cross-sectional worldwide study. The lancet oncology. 2010;11: 1048–1056. 10.1016/S1470-2045(10)70230-8 20952254

[26] Schiffman M, Burk RD, Boyle S, Raine-Bennett T, Katki HA, Gage JC, et al A Study of Genotyping for the Management of Human Papillomavirus-Positive, Cytology-Negative Cervical Screening Results. J Clin Microbiol. 2015;53(1):52–9. 10.1128/JCM.02116-14 25339396 PMC4290932

[27] Wang SS, Gonzalez P, Yu K, Porras C, Li Q, Safaeian M, et al Common genetic variants and risk for HPV persistence and progression to cervical cancer. PLoS One. 2010;5: e8667 10.1371/journal.pone.0008667 20084279 PMC2801608

[28] Roura E, Castellsague X, Pawlita M, Travier N, Waterboer T, Margall N, et al Smoking as a major risk factor for cervical cancer and pre-cancer: results from the EPIC cohort. Int J Cancer. 2014;135: 453–466. 10.1002/ijc.28666 24338632

[29] Bulk S, Bulkmans NW, Berkhof J, Rozendaal L, Boeke AJ, Verheijen RH, et al Risk of high-grade cervical intra-epithelial neoplasia based on cytology and high-risk HPV testing at baseline and at 6-months. Int J Cancer. 2007;121: 361–367. 17354241 10.1002/ijc.22677

[30] Schiffman M, Glass AG, Wentzensen N, Rush BB, Castle PE, Scott DR, et al A long-term prospective study of type-specific human papillomavirus infection and risk of cervical neoplasia among 20,000 women in the Portland Kaiser Cohort Study. Cancer Epidemiol Biomarkers Prev. 2011;20: 1398–1409. 10.1158/1055-9965.EPI-11-0206 21602310 PMC3156084

[31] Koliopoulos G, Arbyn M, Martin-Hirsch P, Kyrgiou M, Prendiville W, Paraskevaidis E, et al Diagnostic accuracy of human papillomavirus testing in primary cervical screening: a systematic review and meta-analysis of non-randomized studies. Gynecol Oncol. 2007;104: 232–246. 17084886 10.1016/j.ygyno.2006.08.053

[32] Solomon D, Davey D, Kurman R, Moriarty A, O'Connor D, Prey M, et al The 2001 Bethesda System: terminology for reporting results of cervical cytology. JAMA. 2002;287: 2114–2119. 11966386 10.1001/jama.287.16.2114

[33] Agorastos T, Koutkias V, Falelakis M, Lekka I, Mikos T, Delopoulos A, et al Semantic integration of cervical cancer data repositories to facilitate multicenter association studies: the ASSIST approach. Cancer Inform. 2009;8: 31–44. 19458792 10.4137/cin.s963 PMC2664695

[34] Bornstein J, Bentley J, Bosze P, Girardi F, Haefner H, Menton M, et al 2011 colposcopic terminology of the International Federation for Cervical Pathology and Colposcopy. Obstet Gynecol. 2012;120: 166–172. 22914406 10.1097/AOG.0b013e318254f90c

[35] Kitchener HC, Almonte M, Thomson C, Wheeler P, Sargent A, Stoykova B, et al HPV testing in combination with liquid-based cytology in primary cervical screening (ARTISTIC): a randomised controlled trial. Lancet Oncol. 2009;10: 672–682. 10.1016/S1470-2045(09)70156-1 19540162

[36] Schmidt CO, Kohlmann T. When to use the odds ratio or the relative risk? Int J Public Health. 2008;53: 165–167. 19127890 10.1007/s00038-008-7068-3

[37] Wilber ST, Fu R. Risk ratios and odds ratios for common events in cross-sectional and cohort studies. Acad Emerg Med. 2010;17: 649–651. 10.1111/j.1553-2712.2010.00773.x 20624147

[38] Bossuyt PM, Reitsma JB, Bruns DE, Gatsonis CA, Glasziou PP, Irwig LM, et al The STARD statement for reporting studies of diagnostic accuracy: explanation and elaboration. Ann Intern Med. 2003;138: W1–12. 12513067 10.7326/0003-4819-138-1-200301070-00012-w1

[39] Sherman ME, Schiffman M, Cox JT. Atypical Squamous Cells of Undetermined Significance/Low-Grade Squamous Intraepithelial Lesion Triage Study G Effects of age and human papilloma viral load on colposcopy triage: data from the randomized Atypical Squamous Cells of Undetermined Significance/Low-Grade Squamous Intraepithelial Lesion Triage Study (ALTS). J Natl Cancer Inst. 2002;94: 102–107. 11792748 10.1093/jnci/94.2.102

[40] Datta SD, Koutsky LA, Ratelle S, Unger ER, Shlay J, McClain T, et al Human papillomavirus infection and cervical cytology in women screened for cervical cancer in the United States, 2003–2005. Ann Intern Med. 2008;148: 493–500. 18378945 10.7326/0003-4819-148-7-200804010-00004

[41] Traut HF, Papanicolaou GN. Cancer of the Uterus: The Vaginal Smear in Its Diagnosis. Cal West Med. 1943;59: 121–122. 18746585 PMC1780413

[42] Peto J, Gilham C, Fletcher O, Matthews FE. The cervical cancer epidemic that screening has prevented in the UK. Lancet. 2004;364: 249–256. 15262102 10.1016/S0140-6736(04)16674-9

[43] Cuzick J, Arbyn M, Sankaranarayanan R, Tsu V, Ronco G, Mayrand MH, et al Overview of human papillomavirus-based and other novel options for cervical cancer screening in developed and developing countries. Vaccine 26 Suppl. 2008;10: K29–41. 10.1016/j.vaccine.2008.06.019 18847555

[44] Arbyn M, Bergeron C, Klinkhamer P, Martin-Hirsch P, Siebers AG, Bulten J, et al Liquid compared with conventional cervical cytology: a systematic review and meta-analysis. Obstet Gynecol. 2008;111: 167–177. 10.1097/01.AOG.0000296488.85807.b3 18165406

[45] Schiffman M, Wentzensen N, Wacholder S, Kinney W, Gage JC, Castle PE, et al Human papillomavirus testing in the prevention of cervical cancer. J Natl Cancer Inst. 2011;103: 368–383. 10.1093/jnci/djq562 21282563 PMC3046952

[46] Saslow D, Solomon D, Lawson HW, Killackey M, Kulasingam SL, Cain J, et al American Cancer Society, American Society for Colposcopy and Cervical Pathology, and American Society for Clinical Pathology screening guidelines for the prevention and early detection of cervical cancer. Am J Clin Pathol. 2012;137: 516–542. 10.1309/AJCPTGD94EVRSJCG 22431528

[47] Naucler P, Ryd W, Tornberg S, Strand A, Wadell G, Elfgren K, et al Human papillomavirus and Papanicolaou tests to screen for cervical cancer. N Engl J Med. 2007;357: 1589–1597. 17942872 10.1056/NEJMoa073204

[48] Ronco G, Giorgi-Rossi P, Carozzi F, Confortini M, Dalla Palma P, Del Mistro A, et al Efficacy of human papillomavirus testing for the detection of invasive cervical cancers and cervical intraepithelial neoplasia: a randomised controlled trial. Lancet Oncol. 2010;11: 249–257. 10.1016/S1470-2045(09)70360-2 20089449

[49] Arbyn M, Ronco G, Anttila A, Meijer CJ, Poljak M, Ogilvie G, et al Evidence regarding human papillomavirus testing in secondary prevention of cervical cancer. Vaccine. 2012;30 Suppl 5: F88–99. 10.1016/j.vaccine.2012.06.095 23199969

[50] Rijkaart DC, Berkhof J, Rozendaal L, van Kemenade FJ, Bulkmans NW, Heideman DA, et al Human papillomavirus testing for the detection of high-grade cervical intraepithelial neoplasia and cancer: final results of the POBASCAM randomised controlled trial. Lancet Oncol. 2012;13: 78–88. 10.1016/S1470-2045(11)70296-0 22177579

[51] Gage JC, Schiffman M, Katki HA, Castle PE, Fetterman B, Wentzensen N, et al Reassurance against future risk of precancer and cancer conferred by a negative human papillomavirus test. J Natl Cancer Inst. 2014;106(8). 10.1093/jnci/dju153 PMC4111283 25038467

[52] Franco EL, Mahmud SM, Tota J, Ferenczy A, Coutlee F. The expected impact of HPV vaccination on the accuracy of cervical cancer screening: the need for a paradigm change. Arch Med Res. 2009;40: 478–485. 10.1016/j.arcmed.2009.06.003 19853188

[53] Cuzick J, Cadman L, Mesher D, Austin J, Ashdown-Barr L, Ho L, et al Comparing the performance of six human papillomavirus tests in a screening population. British journal of cancer. 2013;108: 908–913. 10.1038/bjc.2013.22 23370211 PMC3590662

[54] Heideman DA, Hesselink AT, Berkhof J, van Kemenade F, Melchers WJ, Daalmeijer NF, et al Clinical validation of the cobas 4800 HPV test for cervical screening purposes. J Clin Microbiol. 2011;49: 3983–3985. 10.1128/JCM.05552-11 21880968 PMC3209101

[55] Cox JT, Castle PE, Behrens CM, Sharma A, Wright TC Jr, Cuzick J, et al Comparison of cervical cancer screening strategies incorporating different combinations of cytology, HPV testing, and genotyping for HPV 16/18: results from the ATHENA HPV study. Am J Obstet Gynecol. 2013;208: 184 e181–184 e111. 10.1016/j.ajog.2012.11.020 23174289

[56] Meijer CJ, Berkhof J, Castle PE, Hesselink AT, Franco EL, Ronco G, et al Guidelines for human papillomavirus DNA test requirements for primary cervical cancer screening in women 30 years and older. Int J Cancer. 2009;124: 516–520. 10.1002/ijc.24010 18973271 PMC2789446

[57] Katki HA, Kinney WK, Fetterman B, Lorey T, Poitras NE, Cheung L, et al Cervical cancer risk for women undergoing concurrent testing for human papillomavirus and cervical cytology: a population-based study in routine clinical practice. Lancet Oncol. 2011;12: 663–672. 10.1016/S1470-2045(11)70145-0 21684207 PMC3272857

[58] Wright TC Jr, Massad LS, Dunton CJ, Spitzer M, Wilkinson EJ, Solomon D, et al 2006 consensus guidelines for the management of women with abnormal cervical screening tests. J Low Genit Tract Dis. 2007;11: 201–222. 17917566 10.1097/LGT.0b013e3181585870

[59] Kinney W, Fetterman B, Cox JT, Lorey T, Flanagan T, Castle PE, et al Characteristics of 44 cervical cancers diagnosed following Pap-negative, high risk HPV-positive screening in routine clinical practice. Gynecol Oncol. 2011;121: 309–313. 10.1016/j.ygyno.2010.12.361 21276605 PMC3081890

[60] Kitchener HC, Canfell K, Gilham C, Sargent A, Roberts C, Desai M, et al The clinical effectiveness and cost-effectiveness of primary human papillomavirus cervical screening in England: extended follow-up of the ARTISTIC randomised trial cohort through three screening rounds. Health Technol Assess. 2014;18: 1–196. 10.3310/hta18230 PMC4781243 24762804

[61] Munoz N. Human papillomavirus and cancer: the epidemiological evidence. J Clin Virol. 2000;19: 1–5. 11091143 10.1016/s1386-6532(00)00125-6

[62] Munoz N, Bosch FX, de Sanjose S, Herrero R, Castellsague X, Shah KV, et al Epidemiologic classification of human papillomavirus types associated with cervical cancer. N Engl J Med. 2003;348: 518–527. 12571259 10.1056/NEJMoa021641

[63] Agorastos T, Lambropoulos AF, Sotiriadis A, Mikos T, Togaridou E, Emmanouilides CJ, et al Prevalence and distribution of high-risk human papillomavirus in Greece. Eur J Cancer Prev. 2009;18: 504–509. 10.1097/CEJ.0b013e32832abd5e 19741545

[64] Agorastos T, Chatzistamatiou K, Zafrakas M, Siamanta V, Katsamagkas T, Constantinidis TC, et al Epidemiology of HPV infection and current status of cervical cancer prevention in Greece: final results of the LYSISTRATA cross-sectional study. Eur J Cancer Prev. 2014;23: 425–431. 10.1097/CEJ.0000000000000060 24977385

[65] de Sanjose S, Diaz M, Castellsague X, Clifford G, Bruni L, Munoz N, et al Worldwide prevalence and genotype distribution of cervical human papillomavirus DNA in women with normal cytology: a meta-analysis. Lancet Infect Dis. 2007;7: 453–459. 17597569 10.1016/S1473-3099(07)70158-5

[66] Bruni L, Diaz M, Castellsague X, Ferrer E, Bosch FX, de Sanjose S, et al Cervical human papillomavirus prevalence in 5 continents: meta-analysis of 1 million women with normal cytological findings. J Infect Dis. 2010;202: 1789–1799. 10.1086/657321 21067372

